# Tuning to the Positive: Age-Related Differences in Subjective Perception of Facial Emotion

**DOI:** 10.1371/journal.pone.0145643

**Published:** 2016-01-06

**Authors:** Rochelle Picardo, Andrew S. Baron, Adam K. Anderson, Rebecca M. Todd

**Affiliations:** 1 Department of Psychology, University of British Columbia, Vancouver, British Columbia, Canada; 2 Department of Human Development, Cornell University, Ithaca, New York, United States of America; Brock University, CANADA

## Abstract

Facial expressions aid social transactions and serve as socialization tools, with smiles signaling approval and reward, and angry faces signaling disapproval and punishment. The present study examined whether the subjective experience of positive vs. negative facial expressions differs between children and adults. Specifically, we examined age-related differences in biases toward happy and angry facial expressions. Young children (5–7 years) and young adults (18–29 years) rated the intensity of happy and angry expressions as well as levels of experienced arousal. Results showed that young children—but not young adults—rated happy facial expressions as both more intense and arousing than angry faces. This finding, which we replicated in two independent samples, was not due to differences in the ability to identify facial expressions, and suggests that children are more tuned to information in positive expressions. Together these studies provide evidence that children see unambiguous adult emotional expressions through rose-colored glasses, and suggest that what is emotionally relevant can shift with development.

## Introduction

Facial expressions aid social transactions, allowing individuals to nonverbally communicate their emotions and infer those of others. Across development and cultures, interpretation of such non-verbal signals allows individuals to learn socially acceptable behavior, as well as who and what to approach and avoid [1]. In this context, facial expressions are used as tools for socializing others [1] with smiles signaling approval and reward, and eliciting a reflexive tendency to approach, and angry faces signaling disapproval and potential punishment, and eliciting reflexive avoidance [2–4]. Yet the relative emotional relevance of positive vs. negative emotion may differ according to context and phase of life [5]. That is, distinct from developmental changes in cognitive skill that allows increasing capacity for recognition of facial emotion, there may be developmental shifts in whether expressions of pleasure or anger are more meaningful. Such shifts may in turn be driven by changes in the adaptive benefit of negative vs. positive information. Thus it is critical to identify whether there are developmental differences in tuning to the valence and arousal levels of facial expressions.

Young adults often show an overall bias in attention to negatively valenced stimuli [6,7]. For example, studies in which young adults rate the valence and arousal of both emotional scenes and facial expressions show a pattern in which the more negative the valence, the higher the arousal [8,9] with angry faces rated as higher in arousal than happy faces [9]. Based on these findings, a common assumption in the affective sciences has been that a negative attentional bias is evolutionarily pre-given and constant across the lifespan [6].

### Development of emotion understanding in childhood

One challenge to the view that attention to negative expressions is hardwired comes from research how children learn to understand emotional expressions. For example, *the broad to differentiated hypothesis* proposes that children learn the meaning of specific emotion expressions gradually, in conjunction with behavioral consequences and other contextual cues [10,11]. This hypothesis is supported by evidence that the earliest categorization of facial expressions is based on a crude sorting of facial emotion according to the two basic categories of valence and arousal [10,12]. Differentiation of responses to specific positive and negative expressions is thought to emerge subsequently based on associations between an expression and its behavioral consequences, as well as the good or bad feelings it evokes in the viewer [10,12]. According to this view, sensitivity to happy vs. angry facial expressions may be shaped by a number of environmental factors, and be context-dependent.

### Development of emotion discrimination in childhood

The view that responses to facial emotion are tuned by experience is supported by evidence of a long developmental trajectory for the capacity to categorize or discriminate specific emotions. This trajectory is influenced by the intensity of the expression, and varies between emotion expressions [13–16]. For example, although happy expressions are accurately categorized at adult levels at all but the most subtle intensities by age 5, and all intensities at adult levels by age 7, children under age 10 need higher levels of intensity to recognize angry faces than adults do, and are more likely to categorize angry faces at lower levels of intensity as neutral [16]. Moreover, children with a history of abuse show greater sensitivity to angry faces, further suggesting the environment tunes this capacity [17,18]. Yet age-related differences in the relative subjective relevance of positive vs. negative facial expressions may be distinct from the development of emotion identification skills. Surprisingly little research has addressed the former: Whereas one study examined ratings of subjective arousal and valence for children’s emotional faces in 6–11-year-old children [19], a comparison of children’s and adults’ subjective perceptions of happy vs. threatening expressions, observed on adult faces, is still lacking.

### Age-related differences in bias across the lifespan

Yet another challenge to the assumption that prioritizing negative expressions is hardwired comes from neuroimaging studies examining cognitive changes associated with aging. Here a substantial body of data indicates that affectively-biased attention and concomitant neural responses to positive vs. negative social stimuli may show opposing patterns at different life stages [4,20–22]. For example, unlike young adults, older adults (60+ years) have been found to selectively attend to positive relative to negative facial expressions, and show greater activation in the amygdala, a hub of brain regions sensitive to motivational and affective salience, for positive stimuli [21–23].

Our own recent neuroimaging findings in young children indicate a similar pattern of tuning toward the positive to that observed in older adults, suggesting that perhaps the negative bias observed in young adults is the developmental exception. In an fMRI study in children aged 5–9 years, we observed greater activation in the amygdala when they observed happy relative to angry faces [4], whereas the opposite pattern was observed among young adults (18–35 years) [4]. Among children, amygdala response to angry faces increased with age, suggesting a developmental change in the sensitivity to this particular emotional expression. Finally children, but not young adults, showed greater activation in prefrontal regions necessary for withholding approach responses for positive stimuli, when viewing happy faces [24]. These data suggest developmental differences in the relative emotional relevance of the facial expressions rather than the cognitive capacity to perform the task [24]. The hypothesis that positive information is particularly relevant for children is further supported by studies finding that positive feedback facilitates learning better than negative feedback in children but not in young adults [25–27]. Together, these studies suggest developmental differences in the relevance of positively vs. negatively valenced information. Specifically, children may be particularly sensitive to happy facial expressions on adult faces—potentially because they are perceived as positive feedback.

### The present study

The primary goal of the present study was to employ subjective ratings of valence and arousal to examine differential patterns of emotional relevance between young school-aged children and young adults. Specifically, we wished to examine whether the ‘positivity bias’ we previously observed in patterns of amygdala activation reflected children’s subjective impressions of their own somatic arousal and/or the intensity of the facial expressions—that is whether children “see” happy faces as relatively brighter than adults do, and “feel” them as more arousing. In Studies 1 & 2 we showed children and young adults images of happy and angry faces and had them rate them for subjective impressions of somatic arousal and expression intensity. Based on our previous pattern of neuroimaging findings and convergent behavioural studies, we specifically predicted that children would find happy faces to be more intense and arousing than angry faces, and that young adults would not.

## Study 1

### Methods

#### Participants

28 normally developing children (5–7 years, mean age = 6.3 years, *SD* = 6.6 months, 14 females) were recruited as part of a larger study on emotional biases in development in children aged 4–9 years. Participating families were recruited from a database of interested research participants from the broader Toronto area. Children’s legal guardian(s) were compensated $10 per hour while children received a small toy souvenir for participating. 28 adult participants (19–28 years, mean age = 21.5, *SD* = 2.6 years, 20 female) were recruited for course credit from the University of Toronto. In order to match sample sizes between adults and children, for the present study we selected the youngest 28 children over the age of 5. All participants spoke fluent English and had normal or corrected-to-normal vision. Study 1 was approved by the Humanities and Education REB of the University of Toronto. Parents and adult participants signed informed consent forms and children gave verbal assent prior to participation.

#### Stimuli

Stimuli were 32 angry and happy faces from of the NimStim Face Stimulus Set [28]. Faces were chosen from each of the ethnic face categories in the Nimstim set to be maximally diverse, consistent with the diversity of the families and undergraduates participating in the study. There were equal numbers of male and female faces, which were normed across emotion conditions for intensity and equated for number of mouth open/closed expressions [28]. Stimuli were presented on a computer screen in a two-part task focusing on intensity judgments (Part 1, the ‘It game’) and on subjective responses to the expression (Part 2, the ‘You game’). An experimenter sat beside child participants to explain instructions and advance from one task to the next. Adult participants completed the study on their own in front of a computer.

#### Procedure

Participants completed two tasks, in counterbalanced order. The first task examined the perceived intensity of facial expressions. The second task examined subjective responses to those same expressions. Based on evidence that often-used bipolar scales of valence and arousal (e.g [29]) may be confounded by mixed-valence responses [30], we used separate unipolar scales for each type of judgment (rating of a positive and a negative expression).

#### Intensity Judgments (“It Game”)

For this task, participants were asked to rate the emotional intensity of each facial expression by indicating how glad/mad ‘that person feels.’ In order to control for any potential inconsistencies in children’s emotion ratings [31], participants were asked to rate both how glad and how mad the expression was for both angry and happy faces. To assist participants in understanding the nature of this task, we referred to this as the “It Game” because of its focus on judgments about the emotion of specific faces.

#### Subjective Response Judgments (“You Game”)

Here, participants were asked to rate their perception of their own emotional arousal by indicating how speedy their heart felt when they looked at the face, as well as how sleepy or bored they felt. Finally, participants were asked to rate their emotional responses by indicating how glad and upset they felt looking at each face. We referred to this task as the “You Game” to help participants understand that the focus is on the participant’s own feeling about the facial expression.

The two tasks were structured identically. They began with two practice trials, one displaying a happy and one an angry face, which were identical to test trials except that the text of the test question (eg. “How mad does that person feel?”) appeared on the screen. The faces used in practice trials were not repeated in the test trials. Participants received no feedback on the correctness of their responses in order to emphasize our interest in their subjective, rather than objective, ratings of the faces.

**Fig 1:**
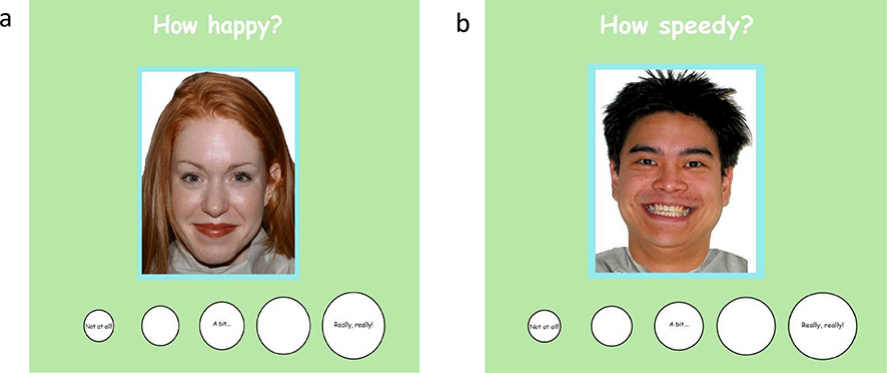
Face Rating Experiment Design. a) The “It” Game was a measure of subjective valence intensity. Participants were asked to point to the circle best describing how happy or angry “the person on the screen feels”. b) The “You” Game was a measure of subjective arousal. Participants were asked to point to the circle best describing the heart speediness/ sleepiness “*you* feel when you look at the person on the screen.” Circles ranged in size from the smallest amount (‘not at all,’ value of 1) to the largest amount (‘really really!’ value of 5).

The test trials consisted of an equal number of randomly presented happy and angry faces paired with questions. Within the It and You games the order of the questions asked of each face was randomized. In each trial, the text of a shortened version of the test question (eg. “How mad?” “How glad?” in the It game and “How speedy?” “How sleepy?” etc. in the You game) appeared near the top of the computer screen, with the stimulus face in the middle and visual rating scale below (Fig 1). The visual rating scale was comprised of five increasingly large circles representing small to large amounts, with the smallest circle labeled “Not at all…” and the largest circle labeled “Really, really!” For child participants a research assistant read the test questions asked children to indicate their response by pointing to one of the circles of the visual rating scale on the screen. Their response was entered by the research assistant using a keyboard to indicate responses from 1 (“Not at all…”) to 5 (“Really, really!”). Adult participants entered their answers. Trials did not advance until a response was entered, and a fixation cross appeared in between trials.

### Results

Repeated measures ANOVAs were performed on ratings of intensity (madness and gladness of faces), somatic feelings of emotional arousal (how speedy and sleepy faces made participants feel), and emotional response (how upset or glad participants felt when they looked at the face). Age group (adult or child) was the between-subjects measure and emotional expression (happy or angry) was the within-subjects measure. See Table 1 for means and S1 File for data for both Studies 1 & 2. All results were Greenhouse-Geisser corrected for violations of sphericity when necessary.

**Table 1:** Study 1 Intensity and Arousal Means.

			Children		Adults	
			*M*	*SD*	*M*	*SD*
**Intensity**		Angry Congruent	4.20	0.62	4.12	0.54
		Happy Congruent	4.58	0.40	3.96	0.45
		Angry Incongruent	1.53	0.37	1.42	0.47
		Happy Incongruent	1.21	0.31	1.22	0.29
**Arousal**		Angry Speedy	3.09	1.16	2.32	0.88
		Happy Speedy	3.24	1.27	2.00	0.70
		Angry Sleepy	2.10	1.18	1.82	0.84
		Happy Sleepy	2.02	1.15	1.77	0.68
**Corrected Intensity (Congruent-Incongruent)**		Angry	2.67	0.85	2.70	0.74
		Happy	3.36	0.51	2.74	0.47
**Corrected Arousal (Speedy -Sleepy)**		Angry	0.99	1.20	0.50	1.04
		Happy	1.23	1.49	0.24	0.81

#### Intensity

In this analysis we separately examined ratings of facial intensity that were congruent and incongruent with the facial emotion (gladness of glad faces, madness of mad faces vs. madness of glad faces and gladness of mad faces). Means are reported in Table 1. For congruent faces, a main effect of age group was observed, *F(*1, 54) = 9.7, *p =* 0.003, ηρ2 = .15, with children rating the faces overall as more intense than adults. This was qualified by an emotional expression by age group interaction, *F(*1, 54) = 11.74, *p =* 0.001, ηρ2 = .18, which revealed that intensity ratings differed by age group. Planned contrasts revealed that children rated happy faces as more intense than angry faces, *p =* 0.001, whereas adults’ intensity ratings did not differ between facial expressions, *p =* 0.16. Moreover, children rated happy faces as more happy than adults did, *p<*0.001, but adults and children rated angry faces as similarly intense *p =* 0.58. We next examined ratings that were incongruent (gladness of mad faces, madness of glad faces). There was no main effect of age group, *p =* 0.34, and no interaction between age group and emotional category, *p =* 0.49. Thus, overall age differences in experienced intensity were observed for congruent expressions only, with children rating happy faces as more intensely happy than angry faces and more intensely happy than adults. In contrast, adults rated happy and angry faces as equally intense, consistent with the norming of the stimuli.

#### Corrected Intensity

**Fig 2:**
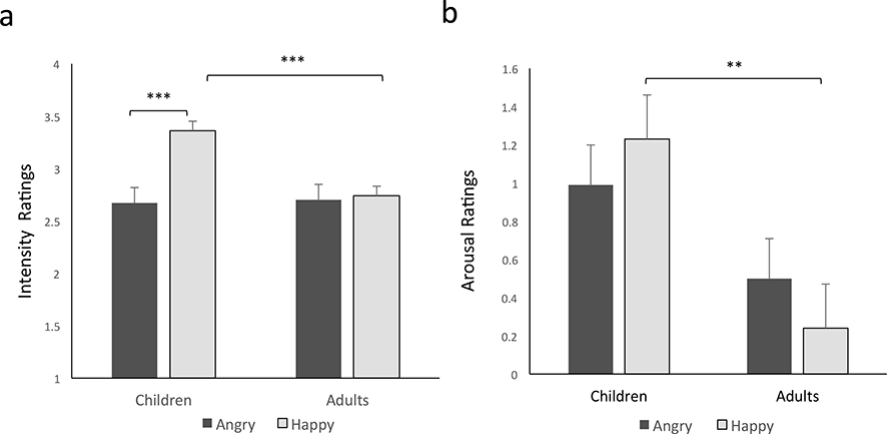
Study 1 a) Corrected intensity ratings (congruent—incongruent trials) show that children rate happy facial expressions as more intense than angry faces, and more intense than do adults. b) Corrected arousal ratings (speediness-sleepiness) show that children rate happy faces as more arousing than adults. ** *p* < 0.005, *** *p* < 0.001.

In this study we told participants the emotional category whether (happiness or angriness) that was to be rated for intensity in each trial. Yet young children are not always reliable in categorization of facial emotions [16], particularly for angry faces, and raw congruent expression ratings may reflect a tendency of children to rate faces as overall more intense (as indeed results of Study 2, below, suggest). To control for potential unreliability and properly compare both studies, we further wanted to examine effects of intensity ratings that were specific to congruent expressions after controlling for ratings of incongruent intensity. To do so we subtracted incongruent ratings (e.g., madness for happy faces) from congruent ratings (e.g., happiness for happy faces) for happy and angry faces separately. ANOVA results showed the main effect of age group found in the analysis of congruent intensity, *F(*1, 54) = 4.59, *p =* 0.04, ηρ2 = .14, indicating that children rated emotional expressions overall as more intense than adults. After controlling for ratings of incongruent faces there was now also a main effect of emotional category, *F(*1, 54) = 11.35, *p =* 0.001, ηρ2 = .17, with overall higher intensity ratings for happy faces. Crucially, as in the analysis of congruent expressions, there was an age group by emotional category interaction, *F(*1, 54) = 9.05, *p =* 0.004, ηρ2 = .14, such that children and adults differed primarily in their intensity ratings of happy faces. Contrasts revealed that, after controlling for incongruent ratings, children rated happy faces as more intense than angry faces, *p<*0.001, and adults’ intensity ratings did not differ between angry and happy, *p =* 0.8. Children differed significantly from adults in rating happy, *p<*0.001, but not angry, *p =* 0.9 faces. Thus, like the ratings for congruent expressions, corrected ratings indicated that, whereas adults experienced angry and happy faces as equally intense, children revealed a positivity bias in subjective experience of the intensity of facial emotion (Fig 2A).

#### Arousal: Speediness and sleepiness ratings

Here we first separately examined effects of age group on the subjective experience of somatic arousal as indexed by speediness and sleepiness ratings. For speediness there was a main effect of age group, *F(*1, 54) = 15.44, *p<*0.001, ηρ2 = .22, such that children rated all facial expressions as eliciting more “speediness” or arousal than their adult counterparts. There was no main effect of emotional category, *p =* 0.43. There was, however, an emotional category by age group interaction, *F(*1, 54) = 5.28, *p =* 0.03, ηρ2 = .09, indicating that children rated happy faces as making their hearts speedier than angry faces and adults rated angry faces as making their hearts speedier than happy faces. For sleepiness ratings, there were no main effects or interactions, *ps*>0.47. Thus, as with intensity, for positive ratings of speediness children again showed higher subjective responses to happy faces and showed the opposite pattern of higher positive speediness responses to angry faces.

#### Corrected arousal

To obtain a measure that was interpretable as positive arousal elicited by each image after controlling for any effects of calmness or boredom, we next subtracted sleepiness ratings from speediness ratings for each face. Again, repeated measures ANOVAs were performed with age group (adult or child) as the between-subjects measure and emotional expression (happy or angry) as the within-subjects measure. As for the raw speediness ratings, results revealed a main effect of age group, *F(*1, 54) = 7.21, *p =* 0.01, ηρ2 = .19, with children rating images as more arousing. There was no main effect of emotional category, *p* = .93. Finally, there was a trend-level interaction between age group and emotional category, *F(*1, 54) = 2.96, *p =* 0.09, ηρ2 = .05, such that children and adults showed opposite tendencies, with children rating happy faces and adults rating angry faces as most arousing. Contrasts revealed that children’s arousal ratings differed from adults’ for happy (*p =* 0.003), but not for angry (*p =* 0.1) faces. In summary, as for the uncorrected speediness ratings children and adults differed in arousal levels experienced in response to happy faces, with children rating them as more arousing than adults, consistent with the positivity bias observed in intensity ratings. In contrast, for arousal adults rated angry faces as more arousing than happy faces, consistent with patterns of negativity bias often reported in young adults (Fig 2B).

#### Emotional response

We examined potential age-related differences in ratings of subjective positive and negative emotional response to facial expression. Here we examined ratings for congruent expressions (how glad faces made them feel, how upset mad faces made them feel). There was a main effect of emotional category, *F(*1, 54) = 21.75, *p<*0.001, ηρ2 = .29, indicating that glad faces elicited more subjective gladness than angry faces elicited feelings of being upset. There was no main effect of age group and no interaction, *ps*>0.18. Thus, in both groups, happy faces elicited more positive emotion than angry faces elicited negative emotion.

Overall these findings revealed a positivity bias in children’s subjective face perception, suggesting that they find happy faces to be more emotionally relevant than do adults. This effect was observed for both intensity of the expression and the perception of somatic arousal elicited by it. The primary goal of Study 2 was to conduct a conceptual replication of the findings in an independent community sample, while adding some modifications and controls to the study. First, in Study 1 there was no neutral face condition to provide a neutral valence baseline. Second, we wished to investigate potential age related differences in emotion-discrimination in two ways: a) By measuring patterns of positive vs. negative bias when discriminating ambiguous facial emotion, and b) by measuring the capacity to discriminate full-intensity happy and angry expressions presented in the face rating task, which allowed us to control for potential group differences in the accuracy of emotion discrimination.

## Study 2

### Methods

#### Participants

26 children (5–7 years, mean age 5.7 years, *SD* = 4.4 months, 14 female) were recruited from a large community-based science center in Vancouver, British Columbia as part of a larger study on development of affective biases. Child participants received a small sticker for their participation. 26 adult participants (19–29 years, mean age 22.8 years, *SD* = 2.9 years, 20 female) were recruited through the undergraduate Human Subjects Pool at the University of British Columbia and participated for course credit. As in Study 1, we selected the youngest 26 children over 5 years for matching sample sizes. All participants spoke fluent English and had normal or corrected to normal vision. Study 2 was approved by the Behavioural Research Ethics Board of the University of British Columbia. Parents and adult participants signed informed consent forms and children gave verbal assent prior to participation.

#### Stimuli

The stimuli used in Study 2 were again taken from the same NimStim Face Stimulus Set [28] used in Study 1, but different faces were selected for this study for the following reason: Given that, even from early in development, responses to emotion other-race faces can differ from responses in own-race faces (e.g., [32]), we wanted to ensure that we chose stimuli representative of the demographics of our Vancouver participants, which was primarily Asian and Caucasian. Thus, equal numbers of Asian (4) and Caucasian (4) female and Asian (2) and Caucasian (2) male faces were utilized. Secondly, Study 2 included a neutral face condition. Because there is evidence that children perceive neutral faces as slightly negative [28,33] we used “calm” faces as neutral stimuli in addition to happy and angry facial expressions. The stimuli were thus comprised of 39 happy, angry and calm faces.

#### Bias Probe Task

We employed a bias probe task to determine potential age-related differences discriminating angry vs. happy faces both when expressions were ambiguous and at full intensity. Participants were presented with randomly shuffled faces drawn from a continuum of unambiguously happy to unambiguously angry faces. To control for own race/other race effects in a population that was primarily either Asian or European-Canadian, two versions of this task, one using a female Caucasian face and one using a female Asian face, were counterbalanced across participants. To create the continua, we used the happy and angry expressions of two female faces (one Caucasian and one Asian) from the NimStim Face Stimulus Set [28] and a morphing software (Abrosoft Fantamorph, Version 5.4.5) that generated a 15-image continuum for each face. Each image was presented randomly three times, and participants were asked in a forced-choice procedure whether each face was happy or angry.

#### Face Rating task

Participants completed the same two tasks outlined in Study 1, but instruction wording and some visual aspects of the tasks differed slightly. Stimuli were presented on a computer in the developmental lab space of the local science centre (for child participants) or a campus lab at UBC (for adult participants). In order to make our tasks more engaging for children, colour was used in screen backgrounds and task instructions to make the experiment more graphically appealing. To better maintain attention, individual components of each trial appeared on the computer screen in succession, with a blue square acting as a fixation cross and stimulus picture frame remaining on the screen throughout. In the “It Game,” the word “happy” was used instead of “glad”. The wording for the “You Game” was identical to Study 1 for children. However, for wording that was more age-appropriate, and for consistency with ratings of arousal in the literature, adults were asked instead to indicate their excitement/anxiety or their feelings of calm/ boredom. Because the previous study did not find any group by emotion differences we did not ask participants to rate their emotional response to faces.

### Results

#### Bias Probe Task

First, in order to examine potential group differences in bias when interpreting emotion on ambiguous faces, we compared the proportion of faces marked happy across all levels of intensity in the bias probe task between children and adults. Here, although data is analyzed across all degrees of morphing between happy and angry faces, it is the 50/50 morphs that reveal differences in bias [34]. T-tests revealed no difference between groups in the proportion of faces rated as happy relative to angry, *t*(50) = 0.03, *p =* 0.97, with both children and adults categorizing faces as happy 54% of the time. Next, in order to examine whether the differences in children’s and adults’ ratings of perceived arousal and intensity was due to differences in ability to discriminate negative relative to positive facial emotion at the intensity used in the face rating task, we looked at accuracy on trials displaying the 100% happy (3) and angry (3) stimuli. T-tests revealed that for angry faces, children were significantly less accurate than adults, *t*(25) = -2.13, *p = 0*.04 (CIs -0.10–-0.002) 7. To ensure that our subsequent analyses were not influenced by these age-related differences in emotion identification ability, we removed children with imperfect accuracy from subsequent analyses, resulting in a final sample of 22 children and 26 adults.

#### Face rating task

Again, repeated measures ANOVAs were performed on intensity (happiness and madness of faces) and arousal (heart speediness and sleepiness of participant). Age group was the between-subjects measure and emotional category was the within-subjects measure. All results were Greenhouse-Geisser corrected for violations of sphericity when necessary. See Table 2 for means.

**Table 2:** Study 2 Intensity and Arousal Means.

			Children		Adults	
			*M*	*SD*	*M*	*SD*
**Intensity**		Angry Congruent	3.98	0.82	4.06	0.88
		Happy Congruent	4.75	0.65	4.38	0.54
		Angry Incongruent	1.75	0.97	1.15	0.24
		Happy Incongruent	1.45	0.90	1.18	0.41
**Arousal**		Calm Speedy	2.84	1.18	1.70	0.76
		Angry Speedy	3.00	1.47	2.92	1.19
		Happy Speedy	3.80	1.55	2.92	1.14
		Calm Sleepy	3.64	0.79	3.74	0.93
		Angry Sleepy	3.63	0.96	1.91	0.68
		Happy Sleepy	1.97	1.26	3.16	0.98
**Corrected Intensity (Congruent- Incongruent)**		Angry	2.26	1.16	2.91	0.99
		Happy	3.30	0.99	3.20	0.62
**Corrected Arousal (Speedy -Sleepy)**		Calm	-0.80	1.24	-2.04	1.48
		Angry	-0.63	1.29	1.01	1.26
		Happy	1.82	2.09	-0.24	1.72

#### Intensity

As in the previous study, we first separately examined intensity ratings for expressions that were congruent and incongruent with the facial emotion. Calm faces were not included in this analysis as the congruent/incongruent distinction made no sense for neutral expressions. There was no main effect of age group, *p =* 0.42. There was a main effect of emotional category, *F(*1, 46) = 19.33, *p<*0.001, ηρ2 = .30, such that happy faces were rated as more intense than angry faces. There was a trend-level emotional category by age group interaction, *F(*1, 46) = 3.28, *p =* 0.08, ηρ2 = .07, such that children rated happy faces as more intense than angry faces, *p<*0.001, and adults rated angry faces as more intense than happy faces at the level of a trend, *p =* 0.06. Moreover, intensity ratings were larger for children than adults for happy faces only, *p =* 0.04. Thus, although the interaction indicating that intensity ratings for expression type varied by age group was trend level, as in Study 1 children showed a clear pattern of rating happy faces as more intense than angry expressions, and more intense than adults.

We next examined ratings for incongruent expressions. Here there was a main effect of age group, *F*(1, 46) = 5.66, *p =* 0.02, ηρ^2^ = .11, such that children rated all faces as more intense than adults did, as well as an emotional category by age group interaction, *F*(1, 46) = 5.03, *p =* 0.03, ηρ^2^ = .10. Contrasts revealed that children rated angry faces as more intensely happy than adults did, *p* = .004, and that they rated angry faces as more intensely happy than they rated happy faces angry. These results suggest that, although ratings of incongruent intensity were low overall (Table 2), children were biased to perceive positive emotion in angry faces despite overall accurate performance on the bias probe in identifying facial emotion.

#### Corrected intensity

**Fig 3:**
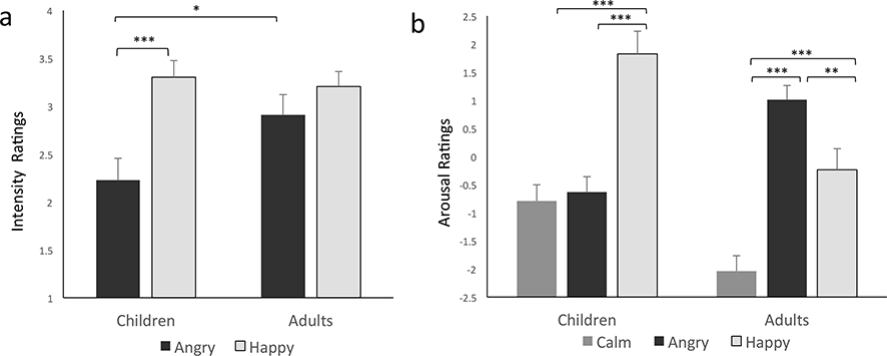
Study 2 a) corrected intensity ratings (congruent—incongruent trials) show that children rate angry faces as less intense than happy faces, and less intense than adults. b) Corrected arousal ratings (speediness-sleepiness) reveal that children and adults differed on ratings of each facial expression, *p*s < 0.05 (for the sake of legibility, the significance of each of these contrasts is *not* illustrated with asterisks). Specifically, children report greater subjective emotional arousal for happy relative to angry faces, while adults show the opposite pattern. *p < 0.05, ** *p* < 0.005, *** *p* < 0.001.

To examine ratings for congruent expresses after correcting for children’s bias to rate more intense happiness in angry faces, we next analyzed difference scores between congruent and incongruent ratings as a measure of intensity for angry and happy faces. Analysis of the difference between congruent and incongruent ratings revealed no main effect of age group, *p =* 0.23. There was a main effect of emotional category, *F(*1, 46) = 25.08, *p<*0.001, ηρ2 = .35, such that happy faces were rated as more intense than angry faces (see Table 2 for means). Crucially, as in Study 1 there was an age group by emotional category interaction, *F(*1, 46) = 8.14, *p =* 0.006, ηρ2 = .15. Once again, planned contrasts revealed that the difference between expressions (Happy > Angry) was significant for children, *p<*0.001, but not for adults, *p* > .18. In this study, children also rated angry faces as less intense than adults did, *p =* 0.03. Thus, after controlling for differences in incongruent intensity ratings, the perceived intensity of happy faces depended on age group. These results conceptually replicated our previous findings that children, but not adults, find happy faces to be more intense relative to angry faces (Fig 3A).

#### Arousal: Speediness and sleepiness ratings

For arousal ratings we included ratings for calm faces in the analysis as a baseline condition. Thus, a 2(children, adults) x 3 (calm, angry, happy) repeated-measures ANOVA was performed on speediness and sleepiness ratings for each emotional category. For speediness ratings, a main effect of age group was observed, *F(*1, 46) = 14.06, *p<*0.001, ηρ2 = .23, such that children rated faces (regardless of expression) as more speedy or exciting than adults. There was a main effect of emotional category, *F(*1, 46) = 8.82, *p<*0.001, ηρ2 = .16, such that angry and happy faces were rated as eliciting more speediness than calm faces. There was no emotional category by age group interaction, *p =* 0.12. Thus in the three-category analysis of positive speediness ratings children rated faces as overall speedier, and all participants rated happy and angry faces as speedier than calm faces.

When we examined ratings of sleepiness, there was no main effect of age group, *p =* .40. There was a main effect of emotional category, *F(*1, 46) = 21.65, *p<*0.001, ηρ2 = .32, such that both age groups rated calm faces as eliciting more sleepiness. This was qualified by an emotional category by age group interaction, *F(*1, 46) = 32.71, *p<*0.001, ηρ2 = .42, such that children reported less sleepiness for happy faces than for the other two emotional categories, *p*s<0.001, while adults reported less sleepiness for angry faces, *ps*<0.001. Children and adults differed significantly in their ratings of sleepiness for angry faces, *p<*0.001, and happy faces *p* = 0.001. Thus, in this study we again observed children’s bias for positive faces relative to adults, but here this pattern was revealed in ratings of greater sleepiness for angry faces, and lower sleepiness for happiness.

#### Corrected arousal

Again, to obtain a measure of positive arousal ratings for each face after controlling for the significant differences in sleepiness ratings, a 2(children, adults) x 3(calm, angry, happy) repeated-measures ANOVA was performed on the difference between speediness and sleepiness ratings for each emotional category. Here, as in Study 1, there was an effect of age group, *F(*1, 46) = 3.65, *p =* 0.02, ηρ2 = .12, such that children rated all facial expressions as more arousing than adults. Here there was also a main effect of emotional category, *F(*1, 46) = 23.29, *p<*0.001, ηρ2 = .34, indicating that calm expressions were rated as less arousing than happy and angry. Again, crucially, there was an emotional category by age group interaction, *F(*1, 46) = 16.89, *p<*0.001, ηρ2 = .27. After subtracting the sleepiness rating from the speediness rating of each face, children and adults differed on arousal ratings for all three facial expressions, *p*s*<* 0.05, with children showing higher corrected arousal ratings for happy and calm faces, and adults showing higher corrected arousal ratings for angry faces. For children, ratings of happy faces differed from those of calm and angry faces, *p<*0.001, whereas calm faces did not differ from angry faces. For adults, all three categories differed from each other, *ps<*0.005. Thus, as in the previous study, children rated happy faces as more arousing than angry faces, whereas adults showed the opposite pattern (Fig 3B). However, as analysis of the uncorrected ratings revealed, in this study the effect was strongly driven by relatively low arousal ratings for angry faces compared to the other two faces conditions—a finding reflected in the expression-specific group differences in sleepiness ratings. This was not due to an inability to recognize angry faces, as all children included in the analysis were 100% reliable in identifying angry expressions. Yet as in Study 1, the overall pattern of results in Study 2 revealed a relative positivity bias in young children’s perception of the intensity and arousal of facial expressions.

## Discussion

In this study we examined children’s subjective perceptions of the valence and arousal of emotional expressions in comparison with those of adults. Results revealed robust findings indicating that, in two separate culturally diverse samples, and with different face stimuli, children rated happy expressions as more intense and more arousing than angry faces, whereas adults rated angry faces as equally intense as, and more arousing than, happy expressions. This finding was not due to differences in the ability to identify facial expressions. This pattern of findings mirrored patterns of brain activation observed in our previous studies [4,24] and provide behavioural evidence in support of the hypothesis that young children see happy faces as more intense and feel more arousal in response to them than adults do. Study 2 also suggested a dampened response to angry faces in children, consistent with a previously-observed pattern of relative amygdala de-activation for angry faces [4]. Our findings suggest that children find happy faces to be more emotionally relevant than angry faces in that they subjectively perceive facial expressions of happiness as being more intensely happy and experience more somatic arousal in response to them than young adults do.

These findings serve to illuminate our own previously-reported neuroimaging findings demonstrating that children show enhanced amygdala activation for happy relative to angry faces [4]. Amygdala activity is thought to reflect detection of the motivational and affective salience of stimuli and deployment of neural resources to systems implicated in attention, memory and motor response [5,35,36]. The pattern of behavioral data reported here, replicated in two independent samples, suggest this pattern of amygdala activation may reflect both greater perceived intensity of the stimulus itself as well as feelings of emotional arousal in response. Importantly, in our previous fMRI study, children viewed expressions posed by their mothers as well as a single matched stranger [4]. The current study further suggests that the enhanced relevance of happy faces observed in children is not restricted to expressions viewed on familiar adult faces. Our neuroimaging study also reported greater activation in the putamen, a key node in mid-brain reward processes[37], for happy faces in children [4]. Such a pattern of putamen activation suggests that happy faces may be experienced more intensely by children in part because they are more rewarding than they are for adults. Future studies can test this hypothesis directly.

A standard view of affect-biased attention has been that humans are evolutionarily predisposed to attend to threatening facial expressions [6]. This view further holds that attentional biases for angry and fearful expressions observed in adults reflect conserved subcortical neural systems, centered on the amygdala, that are hard-wired, fully-automatic, and free from modulation by more deliberate or context-sensitive cortical processes (for a description and critique of this standard view see [38]). This view has been confronted with a number of challenges over the past decade. Behavioral indices of attentional bias to positive vs. negative stimuli are subject to individual differences in temperament, with biases for positive stimuli observed in those high in extraversion and exuberance in adults and children [39,40]. The amygdala, once thought to be an automatic threat detector, has been found to respond equally to positive and negative stimuli [41], and its response to valence can be reversed within minutes depending on task-related goals [42].

Crucially, the direction of bias appears to be modulated by developmental context as well. After controlling for poor health, older adults show behavioural and neural indices of an attentional bias for positive expressions [20–22]. However, positivity biases observed in older adults have been explained in terms of emotion regulation strategies related to the goal of emphasizing the positive in the face of limited time on earth, an explanation consistent with the standard view. Clearly this explanation does not apply to children, and according to the standard view, children should show an even more reliable negativity bias than adults—a prediction in direct opposition to our findings. Taken together these findings suggest a u-shaped curve in the relevance of positive relative to negative facial emotion over the life span, in which the bias toward the negative often observed in undergraduate research participants may be a passing developmental phase rather than a universal human trait. In general, systems tuning us toward the salient may not reflect hard-wired tuning toward specific categories of content but what is salient in a given context. In the present case, developmental shifts in tuning to emotional valence may reflect adaptive responses to the information in children’s environment that is most informative.

Overall, this interpretation is consistent with a body of research examining emotional development in children, which suggests that experience and context play important roles in the development of emotion understanding [10,17]. Previous studies examining facial emotion understanding in children have used a range of methods, including forced choice categorization, free labeling, card sorting, matching faces to stories, matching to sample, and odd man out tasks to test children’s ability to explicitly recognize or discriminate facial expressions (e.g. [11,12,14–17,43]). These studies have found extended and expression-specific developmental trajectories, and suggest a greater reliance on low-level visual features, such as contrast, at younger ages. Overall, the evidence is consistent with the view that early responses to facial expressions are based on subjective responses linked to their perceived consequences [11,43].

Yet although a great deal is known about the development of children’s emotion recognition, little research has probed whether children’s subjective impressions of the intensity of expressions they can recognize, or their own affective responses to the expressions, differ from those of adults. Indeed, to our knowledge, only one study to date has focused on subjective ratings of arousal and valence from children, as is commonly done with adults: Balconi & colleagues [19] employed the self-assessment manikin (SAM), which is commonly used to rate stimulus valence and arousal in adults [29] to collect ratings of valence and arousal for six facial expressions from children aged 6–11. In this study, although both angry and happy faces elicited high levels of subjective and psychophysiological arousal, and heart rate responses did not differ between happy and angry faces, angry faces elicited greater subjective arousal ratings and skin conductance responses.

These conflicting results may be due to a number of methodological differences: First, the age range of the children in the Balconi study was broader and overall older than that examined in the present study, and extended into the pre-pubescent period. The pattern of results we report may be specific to a narrower age-range. Second, there were differences between the subjective rating scales used. The SAM asks observers to rate stimuli on a continuous scale from positive to negative and low to high arousal. In Studies 1 and 2, based on evidence that bipolar scales of valence and arousal may be confounded by mixed-valence responses [30], we predetermined the valence category for each trial and ratings using unipolar scales of positive and negative arousal ratings separately. We also focused our questions about arousal on somatic feelings in order to avoid common linguistic confusions about the meaning of emotional arousal in children—and in particular associations with negative valence. Finally, whereas the Balconi et al. study used photographs of children posing emotional expressions, our study used adult faces. It is possible that children are tuned to very different types of emotional information in peers than in adults. An important question for future research concerns whether children show the same patterns of positivity bias we observed here to expressions on children’s faces.

One discrepancy between Study 1 and Study presented here was that, although children’s arousal ratings for happy faces were consistent across both Study 1 and Study 2, in Study 1 happy and angry faces were rated as equally sleepy, whereas in Study 2 sleepiness ratings were much higher for angry faces. Moreover, in Study 2, children rated angry and calm faces as equally sleepy, as if responses to angry faces were suppressed compared with adults. One possible explanation of this discrepancy is that in Study 1 children focused on speediness ratings in responses to specific faces, and interpreted sleepiness ratings as a rating of a general state of overriding boredom with the experiment. In Study 2 we adjusted the stimulus presentation to make the experience of performing task more engaging, and this may have allowed better interpretation of sleepiness as a response to a specific face. A second possibility is that, when calm faces are included in the design as a baseline condition, in children ratings of angry face arousal are more influenced by the contrasting calm baseline than ratings of happy face arousal. The latter interpretation would suggest that, just as children of this age are more likely to rate lower intensity angry faces as neutral [16], they are also less reliable in rating their own relative arousal in response to angry faces.

This finding of relatively suppressed arousal for angry faces is also consistent with our previous findings that in the youngest children we studied (4–6 years), amygdala activation for angry faces was typically negative in relation to a scrambled face baseline, and amygdala response to angry faces increased linearly with age between the ages of 4 and 9 years [4]. Such a relatively dampened or suppressed response to angry faces may reflect age-related differences in neuromodulator activity. Although little is known about the developmental trajectory of neuromodulator systems in human children, in rats maturation of the norepinephrine system has been linked to acquisition of amygdala-mediated avoidance behaviour [44,45]. Unlike older rat pups, newborn rats fail to show typical expressions of avoidance behavior when they encounter odours associated with foot shock, a behaviour pattern that is thought to facilitate early attachment to caregivers, even in the face of mistreatment [46]. It is thus possible that young children show dampened responses to angry faces to facilitate ongoing attachment to caregivers—though this in turn may be reduced if angry expressions are more informative/experienced as less threatening. Future research can examine whether common genetic variations influencing norepinephrine availability, in interaction with measures of life experience, modulate children’s degree of positivity bias.

Finally, it is important to note that the positivity biases we have observed in subjective reports of arousal and intensity and amygdala activation are not universally observed for other cognitive processes or in other contexts. For example, infants preferentially attend to or remember actors who show antisocial behaviour [47], or do harm to others [48], and infants are more likely to infer that a mechanical object is capable of having a goal if the object was previously observed behaving antisocially but not when it behaved prosocially [49]. Further, school-aged children have been found to interpret ambiguous facial expressions as more negative than adolescents do [50], although we found no difference in bias between young children and adults. An important area for future research therefore will be to further examine the cognitive processes and contexts in which such a bias tuning toward the positive is observed.

In conclusion, we report robust evidence that children may indeed see the world—or at least extreme expressions of adult facial emotion—through rose-colored glasses. Future research can probe the adaptive function of this behavior in specific contexts, how the pattern of bias develops over puberty and adolescence, and genetic and environmental influences on individual differences in tuning toward the positive.

## Supporting Information

S1 File Data for Studies 1 & 2. (XLSX)
